# HPV positive neuroendocrine cervical cancer cells are dependent on Myc but not E6/E7 viral oncogenes

**DOI:** 10.1038/srep45617

**Published:** 2017-04-05

**Authors:** Hang Yuan, Ewa Krawczyk, Jan Blancato, Christopher Albanese, Dan Zhou, Naidong Wang, Siddartha Paul, Faris Alkhilaiwi, Nancy Palechor-Ceron, Aleksandra Dakic, Shuang Fang, Sujata Choudhary, Tung-Wei Hou, Yun-Ling Zheng, Bassem R. Haddad, Yukari Usuda, Dan Hartmann, David Symer, Maura Gillison, Seema Agarwal, Danny Wangsa, Thomas Ried, Xuefeng Liu, Richard Schlegel

**Affiliations:** 1 Department of Pathology, Georgetown University Medical School, Washington DC, 20057, USA; 2 Department of Oncology, Georgetown University Medical School, Washington DC, 20057, USA; 3 College of Pharmacy, King Abulaziz University, Jeddah, Saudi Arabia; 4 Human Cancer Genetics Program and Dept. of Molecular Virology, Immunology and Medical Genetics, Ohio State University Comprehensive Cancer Center, USA; 5 Dept. of Internal Medicine, Ohio State University Comprehensive Cancer Center, Columbus, OH 43210, USA; 6 Cancer Genomics Section, Center for Cancer Research, National Cancer Institute, Bethesda, MD 20892, USA

## Abstract

Using conditional cell reprogramming, we generated a stable cell culture of an extremely rare and aggressive neuroendocrine cervical cancer. The cultured cells contained HPV-16, formed colonies in soft agar and rapidly produced tumors in immunodeficient mice. The HPV-16 genome was integrated adjacent to the Myc gene, both of which were amplified 40-fold. Analysis of RNA transcripts detected fusion of the HPV/Myc genes, arising from apparent microhomologous recombination. Spectral karyotyping (SKY) and fluorescent-*in-situ* hybridization (FISH) demonstrated coordinate localization and translocation of the amplified Myc and HPV genes on chromosomes 8 and 21. Similar to the primary tumor, tumor cell cultures expressed very high levels of the Myc protein and, in contrast to all other HPV-positive cervical cancer cell lines, they harbored a gain-of-function mutation in p53 (R273C). Unexpectedly, viral oncogene knockdown had no effect on the growth of the cells, but it did inhibit the proliferation of a conventional HPV-16 positive cervical cancer cell line. Knockdown of Myc, but not the mutant p53, significantly inhibited tumor cell proliferation. On the basis of these data, we propose that the primary driver of transformation in this aggressive cervical cancer is not HPV oncogene expression but rather the overexpression of Myc.

Cervical cancer is the fourth most common cause of death from cancer in women. In 2012, it was estimated that there were 528,000 worldwide cases of cervical cancer, with 266,000 deaths^1^. Approximately 80% of cervical cancers are squamous cell carcinomas, while 10% are adenocarcinomas and a small number are neuroendocrine carcinomas. In contrast to most cervical cancers, neuroendocrine cancer of the cervix is a highly aggressive malignancy with poor prognosis even in early stages, and it is often undetected by Pap smears due to its downward growth pattern, normal overlying epithelium, and high endocervical location^2^. There is considerable debate concerning the role of HPV in these tumors and no consensus has been reached regarding appropriate therapies. Due to the lack of information on the molecular etiology of these rare tumors, current treatment protocols are modeled on those used for neuroendocrine carcinomas of the lung^3^. Moreover, unlike the well-studied cervical cancer cell lines SiHa (squamous cell carcinoma) and Hela (adenocarcinoma), there is no well-characterized cell line for cervical neuroendocrine carcinoma. In this study, we successfully isolated and propagated the first cancer cell line from a large cell neuroendocrine cervical cancer metastatic to liver. This cell line contained HPV-16 and the viral genome was integrated into host genome adjacent to the *Myc* gene. RNA transcripts were detected which contained a fusion of the *E7*/*Myc* genes and, in contrast of most cervical cancers, the neuroendocrine cells harbored a p53 mutation. Knockdown of *Myc*, but not viral oncogenes or the mutant *p53*, significantly inhibited tumor cell proliferation.

## Results

### Generation of a primary cell culture from a rare and aggressive neuroendocrine cervical cancer

During gynecological examination, a 27-year old female was found to have a 3.5 cm cervical tumor (Supplementary Figure S1). Histology of the tumor revealed a rare large cell neuroendocrine carcinoma of the cervix with high Ki67 staining (93%), suggestive of a highly proliferative malignancy. The tumor stained positively with several neuroendocrine markers including chromogranin A, synaptophysin and somatostatin receptor-2 (Supplemental Table 3). The tumor was initially staged as Ib1 cervical cancer and the patient underwent a radical hysterectomy and lymphadenectomy. At that time of the surgery the primary tumor was 4 cm in diameter, and while the surgical margins were free of tumor, 3 of 16 lymph nodes were positive. She was scheduled for 6 courses of cisplatin and etoposide, which was to be followed by chemo-radiation to the pelvis. However, after the initial day of her 5th course of chemotherapy, the cancer was noted to have progressed rapidly, with at least three left-sided, pelvic masses ranging from 1–3 cm in size. A PET CT scan also revealed three liver metastases. The patient’s condition deteriorated rapidly and she died 3 months later.

**Figure 1:**
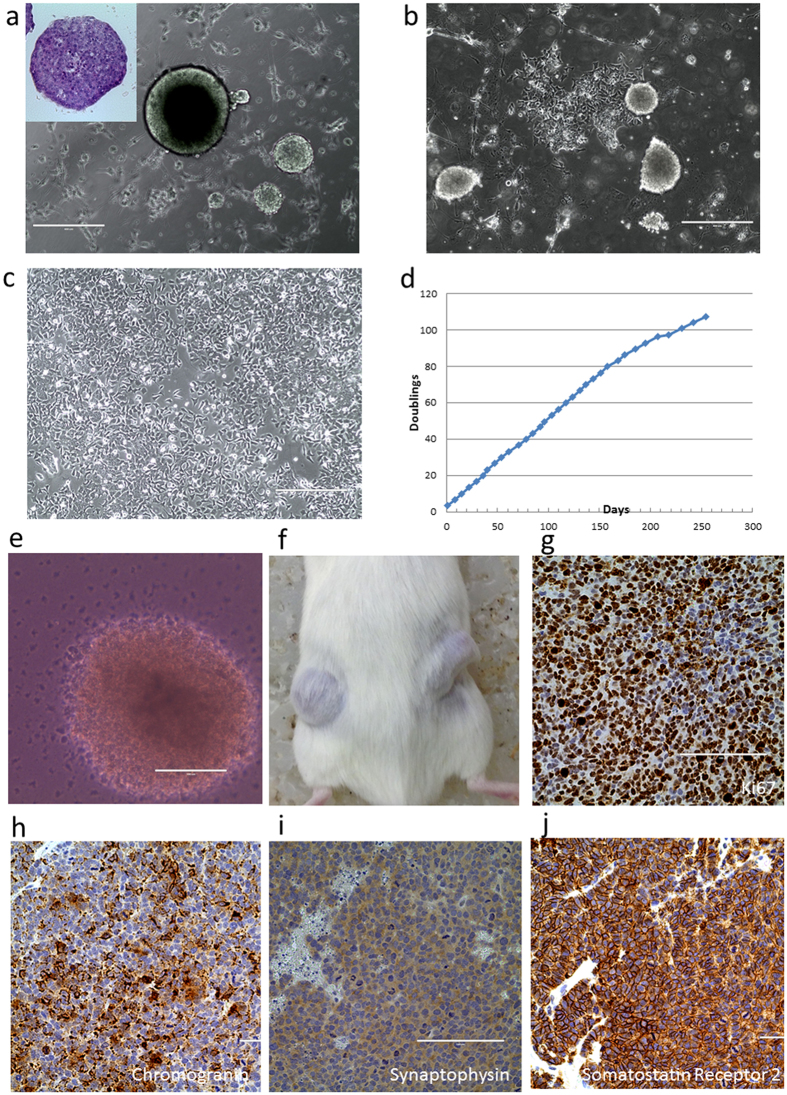
Isolation, propagation and molecular characterization of neuroendocrine cervical cancer cells. (**a**) Phase-contrast micrograph of GUMC-395 cells. Primary cells first grew as aggregates, some of which detached as viable, floating spheroids. An H&E stained section of a floating spheroid is shown in the insert. (**b**) After 2–3 passages, cells remained attached to the substrata and grew as a monolayer (**c**). The best growth condition for monolayer culture was achieved using collagen-coated tissue culture plates and F medium plus ROCK inhibitor, Y-27632. (**d**) Growth curve of GUMC-395 cells in monolayer culture. The transformed phenotype of this cell line was demonstrated by colony formation assay in soft agar (**e**) and by tumor formation in immunodeficient mice (**f**). Immunohistochemistry staining of sections from the xenograft tumors. The tumors showed very high nuclear Ki-67 expression (**g**) and differentiated neuroendocrine markers, including chromogranin (**h**), synaptophysin (**i**) and somatostatin receptor-2 (**j**).

In order to facilitate the analysis of molecular alterations in this lethal neuroendocrine cancer, we established a stable cell culture (GUMC-395) from a liver metastasis using conditional reprogramming^4^,^5^. Initially the cells grew as adherent spheres (Fig. 1a), which soon began to spread outward to form a monolayer (Fig. 1b). An optimum growth condition was defined by screening several substrate and medium formulations (Supplemental Table S1). Formation and proliferation of the monolayer was optimal using a collagen-coated substrate with F medium plus Y-27632 (Fig. 1c). A growth curve of the GUMC-395 culture is displayed in Fig. 1d. The population doubling time was approximately 40 hours. Short Tandem Repeat (STR) profiling of the GUMC-395 cell DNA was identical to that of the patient’s lymphocyte DNA (Supplementary Table S2).

The transformed phenotype of GUMC-395 was demonstrated using cell invasion and migration assays (Supplemental Figures S2 and S3) and anchorage-independent colony formation in soft agar (Fig. 1e). Intriguingly, the cells showed unusual migration into surrounding agar, consistent with their invasive behavior. In xenograft assays, measurable tumors were observed as early as three-weeks post injection into immunodeficient mice. Xenograft experiments were performed three times independently, and a total of 10 out of 10 xenograft sites produced tumors. Similar to the primary tumor (Supplemental Table 3), the xenografts were composed of a high percentage of Ki67-expressing cells (Fig. 1g). Strong expression of chromogranin, synaptophysin, and somatostatin receptor 2 by immunofluorescence and RT-PCR confirmed that the xenografts were of neuroendocrine origin (Fig. 1h to j to j).

### The HPV-16 genome is integrated adjacent to the cellular Myc gene

**Figure 2:**
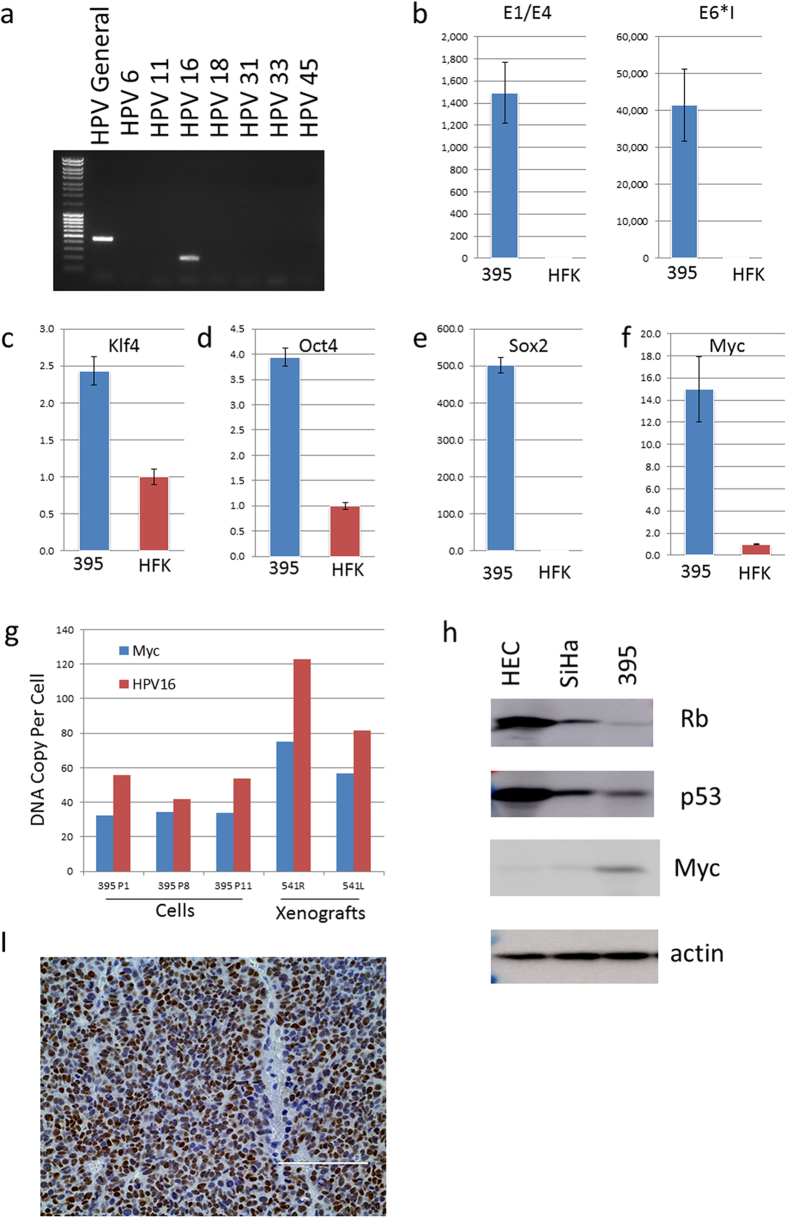
Detection and expression HPV-16 DNA in GUMC-395 and expression of stem cell transcription factors. (**a**) DNA isolated from GUMC-395 was analyzed for the presence of HPVs using HPV general primers and type-specific primers by PCR. (**b**) Spliced HPV-16 RNA E6* and E1^E4 mRNAs were detected using Real-time PCR. (**c–f**) Expression of stem cell reprogramming factors, Klf4, Oct4, Sox2 and Myc were also evaluated by quantitative RT PCR (**c–f**). (**g**) DNA copy numbers of HPV and Myc in cells at different passage and in xenograft tumors were measured by quantitative PCR. (**h**) The levels of Rb, p53 and Myc proteins in GUMC-395 were measured by immunoblots using human keratinoctyes (HFK) and human ectocervical cells (HEC) as controls. (**i**) Immmunohistochecmistry of Myc on a section of GUMC-395 xenograft tumor.

Since HPV is present in squamous carcinoma and adenocarcinoma of the cervix and is postulated to have a role in neuroendocrine cervical cancers, we used general HPV detection primers and HPV type-specific primers to screen the GUMC-395 cultures by PCR (Fig. 2a). The HPV-16 primers generated an appropriate-sized product, which was sequenced and verified to be HPV-16. Evaluation of the viral gene expression in the cells detected spliced viral mRNA for the E6 and E1^E4 (Fig. 2b), reflecting active expression of the HPV viral genome. Further, high expression of stem cell transcription factors, Klf4, Oct4, Sox2 and Myc is consistent with the aggressive and malignant nature of these cells (Fig. 2c–f). Quantitative PCR detected an average of 50 copies of HPV DNA and 30 copies of *Myc* per cell (Fig. 2g). *Myc* gene amplification was further confirmed by CGH analysis (Supplementary Figure S4). Increased Myc protein in GUMC-395 was also confirmed in cell extracts by western blot and in xenograft tumor sections by immunohistochemistry (Fig. 2h and i). Decreased protein levels of RB and p53 were also observed as consequence of HPV-16 infectio*n* (Fig. 2h), consistent with the expression of the E6 and E7 HPV genes.

**Figure 3:**
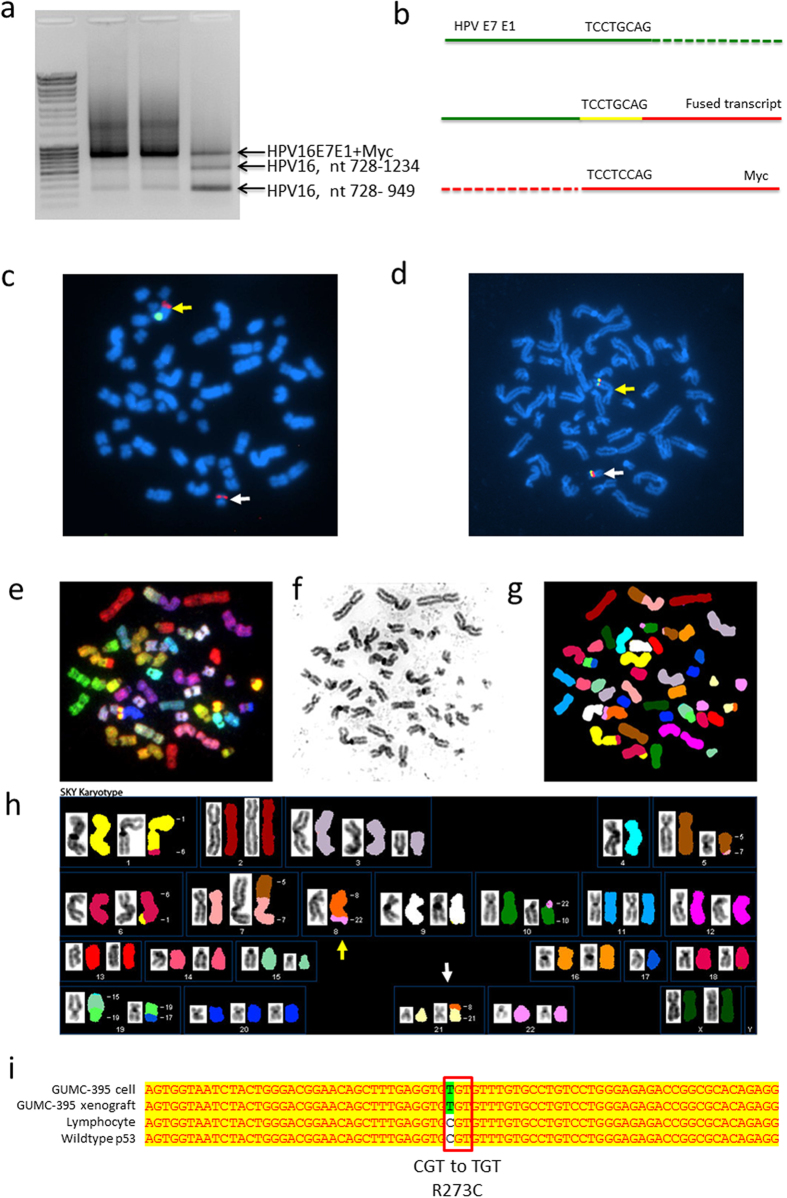
Myc gene amplification and translocation. (**a**) An APOT assay was used to identify the viral-host fusion transcripts. An HPV-Myc fused transcript was detected in all three different passages of GUMC-395. All the transcripts were isolated and sequenced. The sequences of the two small fragments match the HPV-16 nucleotide 728-1234 and 728-949, with no host cell sequence detected. The large fragment contained part of the HPV16 E7E1 sequence and part of the host cell Myc sequence. (**b**) Sequence of the joint sequence between HPV and Myc. The upper line represents the viral HPV16 E7E1 sequence, and the lower line represents the myc sequence. The middle line represents the detected fused transcript, which contained both HPV and Myc sequence. The sequence at the fusion joint was shared between HPV and Myc. (**c**) Fluorescent *in situ* hybridization (FISH) assay using an FITC (green) labeled centromere probe and a spectrum orange (red) labeled c-myc probe. (**d**) Dual labeled FISH assay with spectrum orange (red) labeled Myc probe and FITC (green) labeled HPV-16 probe. MYC and HPV genes are on chromosome 8 (yellow arrow) and 21 (white arrow). (**e**-**h**) A representative graph of Spectral Karyotyping (SKY). (**e**) Chromosome metaphase spread with RGB display colors. (**f**) Inverted-DAPI image of a chromosome metaphase spread. (**g**) Same metaphase spread with classification pseudo-colors. (**h**) Karyotype of the same metaphase spread. Chromosome 8 (yellow arrow) and 21 (white arrow). (**i**) RT-PCR and DNA sequencing revealed that the p53 gene had a somatic mutation, R273C, in GUMC-395 cells. Sequencing of the patient’s lymphocyte DNA showed a wild-type p53 sequence.

Rolling Circle Amplification (RCA) failed to amplify HPV DNA from GUMC-395 (data not shown), suggesting that the HPV-16 genome was integrated into the host genome instead of existing as a free episome. In order to define the integration site of HPV, a 3′RACE protocol (the APOT assay^6^) was used to detect viral-cellular gene fusion transcripts. As shown in Fig. 3a, in addition to the virus-only transcripts, we found an *HPV-Myc* fusion transcript. The junction of the fused transcripts was located at a short, but highly homologous region (a stretch of 8 nucleotides: TCC/GTGCAG) between the HPV and Myc sequences, suggesting that the fusion arose from microhomologous recombination between the *HPV* and *Myc* genes (Fig. 3b)^7^.

### HPV-16 and Myc are amplified and present in chromosomes 8 and 21

Although flow cytometry of propidium iodide-stained nuclei revealed apparent diploid DNA content in GUMC-395 cells (Supplementary Figure S5), karyotyping demonstrated that the cultured cells had many chromosome translocations and copy number variations. The copy number changes included: +2 (43%), +3 or der3 (50%), −4 (80%), −8 (97%), −17 (43%), −19 (50%), +20 (50%), and +22 (40%). The loss of one chromosome 8 in virtually all cancer cells (Fig. 3e) was further validated by the single positive signal (green fluorescence) specific for the centromere of chromosome 8 (Fig. 3c). However, interestingly in this same figure the same chromosome spread, a MYC-specific FISH probe revealed that *Myc* is present in chromosome 8 and is additionally translocated to chromosome 21 (Fig. 3c). Further, as demonstrated in Fig. 3d, the overlapping *Myc* (red) and *HPV* (green) fluorescence signals indicate that HPV-16 was integrated into the host genome adjacent to *Myc* on both of these chromosomes. We further applied spectral karyotyping (SKY)^8^ on GUMC-395 to detect and define the chromosomal translocations observed in the karyotyping analysis (Fig. 3e). The SKY analysis (Fig. 3e–h) revealed several translocations including t(1, 6), t(5, 7), t(10, 22) and t(17–19), in addition to a complex non-homologous translocation involving chromosomes 8, 21 and 22. Part of chromosome 8 was translocated to 21 and part of 22 was translocated to chromosome 8. The t(8:21) translocation was shown in 40 out of 42 metaphase spreads, suggesting that this translocation is an early event in the development/progression of this tumor. In composite, our data demonstrate that GUMC-395 contains HPV-16 that is integrated adjacent to *Myc* on both chromosomes 8 and 21 and that both the HPV-16 genome and *Myc* gene are extensively amplified.

While *p53* is one of the most frequently mutated tumor suppressor genes in cancers^9^, it is only rarely mutated in cervical cancer since the high-risk HPV E6 proteins target p53 for ubiquitin-dependent degradation. In fact, the *p53* mutation occurs in only 5% of cervical cancers^10^ and only in HPV negative cervical tumors^11^,^12^. All HPV-positive cell lines, including Hela, SiHA and Caski, have wild-type p53. We examined the GUMC-395 culture to ascertain whether *p53* was wild-type. Surprisingly, we observed one allele of the *p53* gene in GUMC-395 contained a C to T mutation at nucleotide 817 resulting in a gain-of-function R273C mutation (Fig. 3i). The other allele of *p53* was deleted as determined by CGH (Supplementary Figure S4). This neuroendocrine cell line is the first HPV-positive cell line exclusively harboring a *p53* mutation.

### Proliferation of GUMC-395 cells is independent of the expression of the viral E6/E7 oncogenes

**Figure 4:**
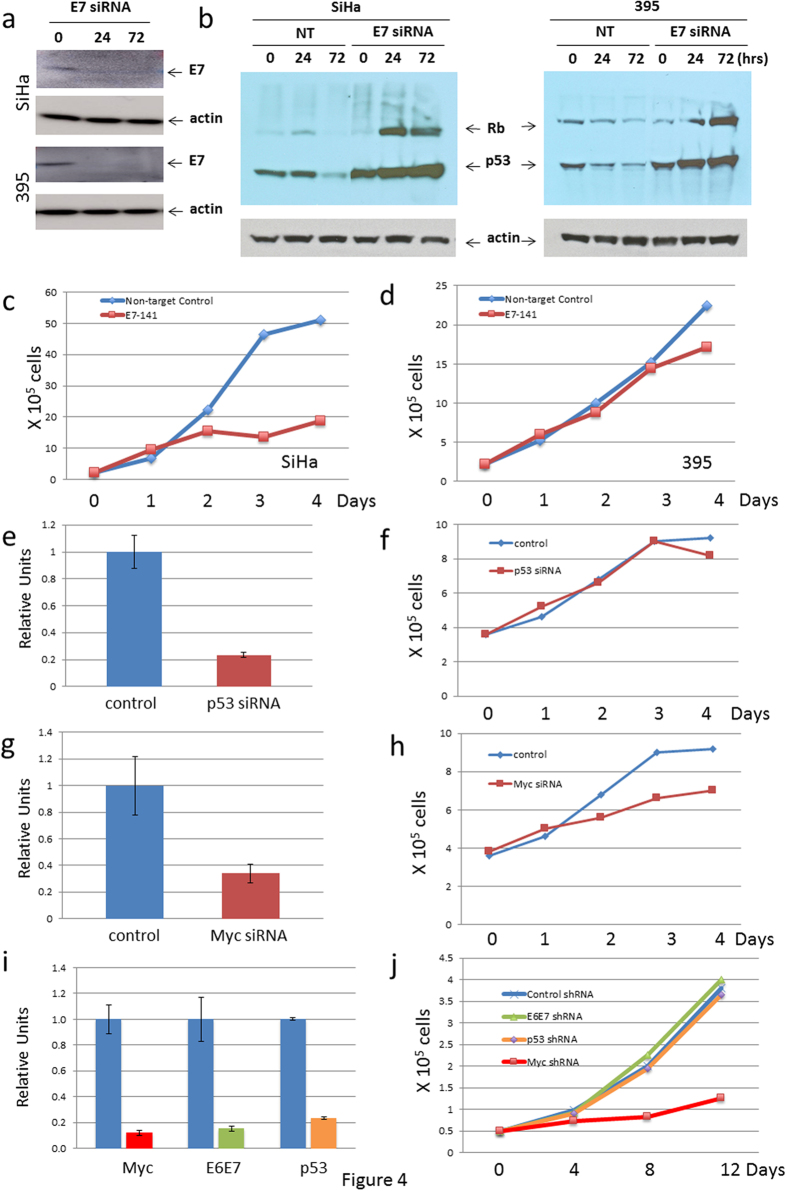
The overexpression of Myc, rather viral oncoproteins E6/E7, is the driving force for tumor cell proliferation. Control non-target RNA and small inhibitory RNA against E7 were transfected into HPV-16 positive SiHa and GUMC-395 cells. (**a** and **b**) Knockdown of E6 and E7 caused a consequential increase in the level of cellular p53 and Rb proteins. The growth of cells was monitored for 96 hours, and cell numbers were counted at 0, 1, 2, 3 and 4 days post transfection. The reduction of E6/E7 and the slowed the proliferation of the SiHa cell line (**c**), but there was no significant impact on the growth of GUMC-395 (**d**). (**e–h**) GUMC-395 cells were transfected with small inhibitory RNA against p53 or Myc using non-target siRNA as a control. The level of p53 (**e**) and Myc (**g**) mRNA were measured by quantitative PCR. The knockdown of Myc (**h**), but not p53 (**g**), inhibited the tumor cell proliferation. (**i** and **j**) GUMC-395 cells were infected with a lentivirus containing non-target control, shRNA targeting E6E7, p53 or Myc. The level of Myc, E6E7 and p53 mRNAs was measured by quantitative PCR (**i**). The growth of cells was monitored for 12 days (**j**). Knockdown of Myc, but not the mutant p53 or viral E6/E7, significantly inhibited tumor cell proliferation.

It is well established that the proliferation and survival of cervical cancer cell lines require the continued expression of the HPV *E6* and *E7* genes, which respectively lead to the degradation of the cellular p53 and Rb proteins^13^. For example, established cervical cancer cell lines such as HeLa, SiHa and CaSki^14^,^15^,^16^,^17^, as well as primary cervical cancer lines established directly from fresh biopsies^18^, remain reliant on the HPV *E6*/*E7* genes for continued proliferation. To evaluate if the unusual GUMC-395 cell culture shared this same viral oncogene dependence, we used both siRNA and shRNA to knockdown E6 and E7 expression and evaluate whether cell proliferation was affected. Due to the splicing pattern of the *E6*/*E7* genes in HPV-16, knockdown of E7 will reduce the expression of both the *E6* and *E7* genes. We documented that the transiently transfected E7 siRNA reduced both E6 and E7 mRNA in a 4-day experiment, and, more importantly, caused a consequential dramatic increase in the level of cellular p53 and Rb proteins in the HPV-16 positive SiHa cell line (Fig. 4a) and in the GUMC-395 cells (Fig. 4b). While the reduction of E6/E7 and the resultant increase in p53 and Rb greatly slowed the proliferation of the SiHa cell line (Fig. 4c), there was no significant impact on the growth of GUMC-395 cells (Fig. 4d). This experiment was repeated two additional times, and the same effect was observed. To further evaluate whether the GUMC-395 cells remained resistant to E6/E7 knockdown beyond 4-day time points, we utilized shRNA lentiviruses that targeted E6/E7 expression to generate stable cell cultures. As shown in Fig. 4i, the lentivirus infected cells had greatly reduced expression of E6/E7 but this had no effect on GUMC-395 proliferation (Fig. 4j), even at 14 days. This is the first example of an HPV-positive cervical cancer cell line being independent of E6/E7 expression.

Growing evidence demonstrates that some mutant p53 proteins exhibit a gain-of-function phenotype that can contribute to malignant progression^19^. More specifically, recent studies indicate that the R273C p53 mutation can enhance cell proliferation^20^. To evaluate whether the R273C p53 mutation contributed to the proliferation of GUMC-395, we used siRNA to reduce cellular p53 levels (Fig. 4e). However, despite reducing p53 mRNA expression by approximately 80%, there was no effect on the growth of the GUMC-395 cells or the control SiHa cells (Fig. 4f). In contrast to the lack of effect of p53 knockdown, siRNA against Myc significantly reduced Myc mRNA expression (Fig. 4g) and inhibited the proliferation of GUMC-395 cell cultures in a 4-day experiment (Fig. 4h). To determine if these findings would be validated with longer-term knockdown, we used shRNA lentivirus to examine cell proliferation up to 12 days. In these extended experiments we confirmed that knockdown of Myc, but not the mutant p53 or viral E6/E7, significantly inhibited tumor cell proliferation (Fig. 4i and j). Thus, both siRNA and shRNA data indicate that the proliferation of GUMC-395 is independent of E6/E7 expression and resistant to the resultant increase in cellular p53 and Rb. One alternative interpretation of these results is that the presence of the ROCK inhibitor, Y-27632, in the medium of GUMC-395 cells might bypass the requirement for E6/E7 expression. To address this possibility, we transduced SiHa cells in medium containing the same concentration of Y-27632 that is used to propagate the GUMC-395 cells (Supplemental Figure 6). If Y-27632 were able to bypass the inhibitory effects of siRNA to E6/E7 (either via modulating HPV or Myc), then we would expect the SiHa cells to grow at the same rate as control-transduced cells. However, we found that, similar to Fig. 4c, the SiHa cells were indeed inhibited by the siRNA. Y-27632 does not bypass the requirement for continued expression of E6/E7 and we can conclude that the GUMC-395 cell line, in contrast to standard HPV cell lines such as SiHa, does not require E6/E7 protein to proliferate *in vitro*. While our studies indicate that Myc is essential for HPV positive GUMC-395 cell proliferation, clearly we cannot claim tumor cell specificity since Myc is also required for normal cell proliferation. However, our data does suggest that new approaches for controlling Myc overexpression might be useful in treating this tumor type.

## Discussion

The molecular etiology of neuroendocrine cervical cancer is poorly understood due to the lack of well-defined cell lines. We have successfully generated and propagated a culture (GUMC-395) of an HPV-16-positive, large cell neuroendocrine cervical cancer that was metastatic to the liver. GUMC-395 cells expressed the HPV-16 E6 and E7 oncogenes, but unlike all other HPV-positive cervical cancers, their proliferation was independent of these viral proteins. Indeed, it appears that the overexpression of Myc is the predominant driver of transformation in GUMC-395. We presume that the overexpression of the Myc protein in the cell line results from the amplification of the Myc gene. The primary tumor also showed dramatic overexpression of the Myc gene, suggesting that this event was not the consequence of cell line establishment. However, since we did not evaluate Myc gene copy number in the primary tumor, we cannot conclude that the overexpression of Myc protein in the primary tumor was the consequence of gene amplification.

The *Myc* proto-oncogene has been shown previously to be deregulated in many types of tumors including breast, colon, small-cell lung carcinomas, osteosarcomas, glioblastomas, melanoma, myeloid leukemias, and cervical cancers (reviewed in ref. 21). Studies have shown that 30% of all HPV-18 integrations occur within the chromosomal band 8q24 near the *Myc* proto-oncogene, whereas HPV-16 integration occurs near Myc at a much lower rate^22^,^23^,^24^. Interestingly, integration of HPV into the *Myc* region strongly correlates with high levels of Myc expression^7^,^23^. In earlier studies short overlaps between HPV and genomic sequences were observed^25^,^26^. A recent study on cervical squamous carcinoma and adenocarcinoma has shown a significant enrichment of microhomologies (MHs) between the human genome and the HPV genome at or near integration breakpoints^7^. Our data suggest that, in the GUMC-395 culture, MH-mediated DNA repair pathways and MH-mediated, break-induced replication might be also be involved in HPV integration into the cellular genome. The GUMC-395 cells now provide the first *in vitro* platform for analyzing the molecular mechanism of HPV integration and an opportunity to identify potential therapies for this rare, aggressive and rapidly lethal cervical cancer.

It is well documented that p53 mutations are very rare in cervical cancers^12^,^27^,^28^ and the analysis of cell lines derived from most HPV-positive cervical cancers has shown the p53 gene to be wild-type. p53 mutations have occasionally been shown in HPV-negative cervical tumors, although at a much lower rate compared to other types of cancers^29^. The GUMC-395 cervical cell line described in this study is the first to harbor a *p53* mutation. Even more interesting, the mutation is predicted to have a gain-of-function phenotype. It is unclear how this specific mutation might contribute to the current growth properties of the cell line, especially since the presence of an active HPV E6 oncogene greatly reduced the level of p53 protein in the cells. In addition, further reduction of p53 by siRNA also had no effect on cell proliferation. However, we do not know the chronology of the genetic changes during the evolution of this tumor and it is possible that an early mutation in P53 was critical in the initiation of neoplasia. Interestingly, it was shown previously that a mis-sense mutation in p53 (codon 245) was present in an HPV-18 positive small cell neuroendocrine carcinoma of the cervix^30^. The possibility exists that tumor suppressor proteins such as p53 have somewhat different activities in cervical neuroendocrine cells and cervical squamous cells.

## Materials and Methods

### Ethics Statement

The patient was enrolled into study number 03-C-0277 at the National Cancer Institute. The study was approved by National Cancer Institute Institutional Review Board. Informed, written consent was obtained from participant prior to any study procedures. All experiments were performed in accordance with protocol relevant guidelines and regulations.

### Tissue processing, cell isolation and propagation

Tumor samples were obtained with the informed consent of the patient according to a National Cancer Institute IRB protocol. Cancer cells were isolated from the patient’s metastatic liver biopsy, then propagated according to the CRC method described previously^4^. The stable cell culture was termed GUMC-395. An optimum growth condition was defined by screening several cell culture coating substrates and medium formulations (Supplemental Table 1). All media were used in the combination of coated flasks with or without 1 mg/ml gelatin (Sigma), 1 mg/ml rat tail collagen type I (Corning) or matrigel (Corning). The optimal culture conditions were the CRC F medium supplemented with 10 μM Y-27632 (FY medium) in a collagen type I-coated flask. For quality control a STR analysis was performed on early and late passages, as well as on lymphocyte DNA from the patient.

### Xenograft Assays

Exponentially tumor cells (1 × 10^6^) were trypsinized, dispersed into single cells, and suspended in 200 μL of Matrigel HC (BD-growing Biosciences). The Matrigel-suspended cells were injected subcutaneously into the left and right flanks of 6-week-old male mice with severe combined immunodeficiency (Taconic, Germantown, NY). The growth of xenografts was measured weekly with calipers. Animals were housed at the Georgetown University animal care facility according to institutional guidelines. Animal protocol #14-033-100171 was approved by Institutional Animal Care and Use Committee (IACUC) at Georgetown University. All experiments were performed in accordance with the protocol relevant guidelines and regulations.

### Histological staining, immunohistochemistry and immunofluorescence staining

Cells or xenografts were fixed in formalin, paraffin embedded, sectioned, and stained with hematoxylin and eosin (H&E) using standard procedures. Sections were deparaffinized and rehydrated for fluorescent immunolabeling using the following antibodies: anti-synaptophysin (Millipore MAB368, 1:100), anti-chromogranin (Abcam ab15160, 1:100), anti-somatostatin (Abcam #ab134152, 1:100), anti-Myc (Abcam ab32072, 1:100), anti-Ki-67 (Abcam ab15380, 1:100), anti-progesterone receptor (Santa Cruz Biotechnology sc-538, 1:200), anti-estrogen receptor (Santa Cruz Biotechnology sc-8002, 1:100), anti-oxytocin (Santa Cruz Biotechnology sc-33209, 1:100), anti-prolactin (Santa Cruz Biotechnology sc-46698, 1:100), anti-growth hormone (GH) (Santa Cruz Biotechnology sc-374266, 1:100), anti-follicle-stimulating hormone (FSH sc-374452, 1:100) (Santa Cruz Biotechnology), anti-thyroid-stimulating hormone (TSH) (Abcam ab6071, 1:100), anti-vasopressin (Abcam ab39363, 1:100), anti-insulin (Cell Signaling #4590, 1:200), anti-glucagon (Cell Signaling #2760, 1:200), anti-trefoil factor-1 (TFF-1) (Leica Microsystems NCL-L-TTF-1, 1:200) and anti-neural cell adhesion molecule (NCAM) (Abcam ab133345, 1:100).

### DNA isolation, cloning and sequencing

Total DNA was isolated from the patient’s cultured cells or xenografts using DNeasy Blood & Tissue Kit (Qiagen). The DNA preparation was subjected to polymerase chain reaction (PCR) analysis using HPV general primers or HPV type-specific primers according to protocol previously described^5^. Primer sequences: HPV6, 5′-TAGTGGGCCTATGGCTCGTC-3′ and 5′-TCCATTAGCCTCCACGGGTG-3′; HPV11, 5′-GGAATACATGCGCCATGTGG-3′, 5′-CGAGCAGACGTCCGTCCTCG-3′; HPV16, 5-TTATGAGCAATTAAATGACAGCTCAG-3, and 5′-TGAGAACAGATGGGGCACACAAT-3′; HPV18, 5′-CGACAGGAACGACTCCAACGA-3′ and 5′-GCTGGTAAATGTTGATGATTAACT-3′; HPV31, 5′-AGCAATTACCCGACAGCTCAGAT-3′ and 5′-GTAGAACAGTTGGGGCACACGA-3′; HPV33, 5′-ACTGACCTAYACTGCTATGAGCAA-3′and 5′-TGTGCACAGSTAGGGCACACAAT-3′; HPV45, 5′-TTAAGGACAAACGAAGATTTCACA-3′ and 5′-ACACAACAGGTCAACAGGATCTAA-3′.

### mRNA Quantification

Total RNA was isolated using QIAGEN RNeasy Mini Kit (Cat#: 74104) according to the manufacturer’s protocol. cDNA synthesis was performed with (Cat#: 215011) FastLane Cell cDNA Kit. Quantitative real-time RT-PCR (RT-qPCR) was performed as described previously^4^,^5^ using iCycler MyiQ and iQ SYBR Green SuperMix (Bio-Rad Laboratories, Hercules, CA). The following forward and reverse primers were used:

E1E4, 5′-TGGCTGATCCTGCAGCAGC-3′ and 5′-AGGCGACGGCTTTGGTATG-3′;

E6*I, 5′-ACAGTTACTGCGACGTGAGATG -3′, and 5′–TTCTTCAGGACACAGTGG-3′;

unspliced E6, 5′-CAACAAACCGTTGTGTGAT-3′, and 5′-CGTGTTCTTGATGATCTGC-3′;

E7: 5′-ATGCATGGAGATACACCTAC-3′ and 5′-CATTAACAGGTCTTCCAAAG-3′;

Myc, 5′-ACCACCAGCAGCGACTCTGA-3′ and 5′-TCCAGCAGAAGGTGATCCAGACT-3′, probe 5′-GAGGCAGGCTCCTGGCAAAAGGTC-3′;

OCT4: 5′-GGGTTTTTGGGATTAAGTTCTTCA-3′ and 5′-GCCCCCACCCTTTGTGTT -3′

SOX2: 5′-CAAAAATGGCCATGCAGGTT-3′ and 5′-AGTTGGGATCGAACAAAAGCTATT-3′

KLF4: 5′-AGCCTAAATGATGGTGCTTGGT-3′ and 5′-TTGAAAACTTTGGCTTCCTTGTT-3′;

p53: 5′-GGAGCCGCAGTCAGATCCTA-3′ and 5′-GGGGACAGAACGTTGTTTTC-3′; GAPDH, 5′-TCTCCTCTGACTTCAACAGC-3′ and 5′-GAAATGAGCTTGACAAAGTG-3′;

hTERT mRNA was quantitatively measured using published primers and methods^1^. All data were normalized to levels of GAPDH.

### DNA Copy number using quantitative PCR

TaqMan real-time quantitative PCR was performed on a Bio-Rad iCyclerMyiQ, using primers and probes for the quantitation of HPV16 E7 (sense primer, 5′-AGG AGG ATG AAA TAG ATG GTC C-3′; anti-sense primer, 5-CTT TGT ACG CAC AAC CGA AGC-3′). Myc copy number was measured by human Myc TaqMan CPopy Number Assay (Thermo Fisher # Hs01764918_cn. TaqMan^®^ Copy Number Reference Assay human RNase P (Thermo Fisher #4403328) was used as an endogenous reference in each reaction. Realtime PCR reactions were done in triplicate for all samples. The levels of HPV DNA were analyzed using iQ5 software with the normalized expression ({Delta}{Delta}CT) method according to the manufacturer’s (Bio-Rad’s) guidelines.

### Cell transfection with siRNA and cell infection with shRNA

Cells were transfected according to manufacturer’s instructions using Lipofectamine RNAiMAX (Invitrogen) and 9 nM siRNA. To control for non-specific siRNA effects, a pool of non-targeting control siRNAs (ON-TARGET plus non-targeting, Thermo Scientific) was used, also at 9 nM. siRNAs for E7 (GGACAGAGCCCAUUACAAU), p53 and Myc were purchased from Dharmacon. Cell numbers were counted every 24 hours for up to 4 days. Lentiviruses with Myc shRNA, p53 shRNA, E6E7 shRNA or GFP shRNA were purchased from Santa Cruz, GUMC-395 cells were infected according to the manufacturer’s protocol. 5 × 10^4^ infected cells were plated and numbers of the attached viable cells were counted every 4 days, the curve was plotted as cell numbers versus days after plating.

### Spectral Karyotyping (SKY) and Analysis

Preparation of SKY probes, slide pre-treatment, slide denaturation, detection, and imaging have been described previously^31^. Protocols can be accessed at: http://www.riedlab.nci.nih.gov/protocols. Metaphase chromosome suspensions were prepared by treating cells with a hypotonic solution (0.075 M KCl), followed by methanol: acetic acid (3:1, vol/vol) fixation. The suspension was then dropped onto slides using a Thermatron™ to control humidity. The slides were aged at 37 °C for approximately one week prior to hybridization. Chromosome preparations were hybridized with SKY probes (prepared in-house) for 72 hours. Slides were imaged for SKY analysis using a Leica DMRXE microscope (Leica, Germany) equipped with DAPI and SKY™ filters (Chroma, Bellows Falls, VT), a Xenon lamp, and Spectracube™ (Applied Spectral Imaging, Vista, CA). Spectrum-based classification and analysis of the fluorescent images (SKY) was achieved using SkyViewTM software (Applied Spectral Imaging). Approximately 15–20 metaphase spreads were acquired for SKY analysis for each cell line and scored for numerical and structural chromosomal aberrations according to established human chromosome nomenclature rules from ISCN (2009)[2].

## Additional Information

**How to cite this article**: Yuan, H. *et al*. HPV positive neuroendocrine cervical cancer cells are dependent on Myc but not E6/E7 viral oncogenes. *Sci. Rep.* **7**, 45617; doi: 10.1038/srep45617 (2017).

**Publisher's note:** Springer Nature remains neutral with regard to jurisdictional claims in published maps and institutional affiliations.

## Supplementary Material

Supplementary Information

## References

[b1] Arteaga C. L. . AACR Cancer Progress Report 2014. Clin Cancer Res 20, S1–S112 (2014). 25228531 10.1158/1078-0432.CCR-14-2123 PMC4666597

[b2] McCusker M. E., Cote T. R., Clegg L. X. & Tavassoli F. J. Endocrine tumors of the uterine cervix: incidence, demographics, and survival with comparison to squamous cell carcinoma. Gynecol Oncol 88, 333–339 (2003). 12648583 10.1016/s0090-8258(02)00150-6

[b3] Gardner G. J., Reidy-Lagunes D. & Gehrig P. A. Neuroendocrine tumors of the gynecologic tract: A Society of Gynecologic Oncology (SGO) clinical document. Gynecol Oncol 122, 190–198 (2011). 21621706 10.1016/j.ygyno.2011.04.011

[b4] Liu X. . ROCK inhibitor and feeder cells induce the conditional reprogramming of epithelial cells. Am J Pathol 180, 599–607 (2012). 22189618 10.1016/j.ajpath.2011.10.036 PMC3349876

[b5] Yuan H. . Use of reprogrammed cells to identify therapy for respiratory papillomatosis. N Engl J Med 367, 1220–1227 (2012). 23013073 10.1056/NEJMoa1203055 PMC4030597

[b6] Klaes R. . Detection of high-risk cervical intraepithelial neoplasia and cervical cancer by amplification of transcripts derived from integrated papillomavirus oncogenes. Cancer Res 59, 6132–6136 (1999). 10626803

[b7] Hu Z. . Genome-wide profiling of HPV integration in cervical cancer identifies clustered genomic hot spots and a potential microhomology-mediated integration mechanism. Nat Genet 47, 158–163 (2015). 25581428 10.1038/ng.3178

[b8] Padilla-Nash H. M., Barenboim-Stapleton L., Difilippantonio M. J. & Ried T. Spectral karyotyping analysis of human and mouse chromosomes. Nat Protoc 1, 3129–3142 (2006). 17406576 10.1038/nprot.2006.358 PMC4772431

[b9] Muller P. A. & Vousden K. H. Mutant p53 in cancer: new functions and therapeutic opportunities. Cancer Cell 25, 304–317 (2014). 24651012 10.1016/j.ccr.2014.01.021 PMC3970583

[b10] Ojesina A. I. . Landscape of genomic alterations in cervical carcinomas. Nature 506, 371–375 (2014). 24390348 10.1038/nature12881 PMC4161954

[b11] Crook T., Wrede D. & Vousden K. H. p53 point mutation in HPV negative human cervical carcinoma cell lines. Oncogene 6, 873–875 (1991). 1646990

[b12] Scheffner M., Munger K., Byrne J. C. & Howley P. M. The state of the p53 and retinoblastoma genes in human cervical carcinoma cell lines. Proc Natl Acad Sci USA 88, 5523–5527 (1991). 1648218 10.1073/pnas.88.13.5523 PMC51909

[b13] Moody C. A. & Laimins L. A. Human papillomavirus oncoproteins: pathways to transformation. Nat Rev Cancer 10, 550–560 (2010). 20592731 10.1038/nrc2886

[b14] Francis D. A., Schmid S. I. & Howley P. M. Repression of the integrated papillomavirus E6/E7 promoter is required for growth suppression of cervical cancer cells. J Virol 74, 2679–2686 (2000). 10684283 10.1128/jvi.74.6.2679-2686.2000 PMC111757

[b15] Goodwin E. C. & DiMaio D. Repression of human papillomavirus oncogenes in HeLa cervical carcinoma cells causes the orderly reactivation of dormant tumor suppressor pathways. Proc Natl Acad Sci USA 97, 12513–12518 (2000). 11070078 10.1073/pnas.97.23.12513 PMC18795

[b16] Jiang M. & Milner J. Selective silencing of viral gene expression in HPV-positive human cervical carcinoma cells treated with siRNA, a primer of RNA interference. Oncogene 21, 6041–6048 (2002). 12203116 10.1038/sj.onc.1205878

[b17] Parish J. L. . E2 proteins from high- and low-risk human papillomavirus types differ in their ability to bind p53 and induce apoptotic cell death. J Virol 80, 4580–4590 (2006). 16611918 10.1128/JVI.80.9.4580-4590.2006 PMC1472007

[b18] Magaldi T. G. . Primary human cervical carcinoma cells require human papillomavirus E6 and E7 expression for ongoing proliferation. Virology 422, 114–124 (2012). 22056390 10.1016/j.virol.2011.10.012 PMC3229657

[b19] Wang G. & Fersht A. R. Propagation of aggregated p53: Cross-reaction and coaggregation vs. seeding. Proc Natl Acad Sci USA 112, 2443–2448 (2015). 25675527 10.1073/pnas.1500262112 PMC4345553

[b20] Vaughan C. A. . p53 mutants induce transcription of NF-kappaB2 in H1299 cells through CBP and STAT binding on the NF-kappaB2 promoter and gain of function activity. Arch Biochem Biophys 518, 79–88 (2012). 22198284 10.1016/j.abb.2011.12.006 PMC3272778

[b21] Pelengaris S., Khan M. & Evan G. c-MYC: more than just a matter of life and death. Nat Rev Cancer 2, 764–776 (2002). 12360279 10.1038/nrc904

[b22] Ferber M. J. . Preferential integration of human papillomavirus type 18 near the c-myc locus in cervical carcinoma. Oncogene 22, 7233–7242 (2003). 14562053 10.1038/sj.onc.1207006

[b23] Peter M. . MYC activation associated with the integration of HPV DNA at the MYC locus in genital tumors. Oncogene 25, 5985–5993 (2006). 16682952 10.1038/sj.onc.1209625

[b24] Schmitz M., Driesch C., Jansen L., Runnebaum I. B. & Durst M. Non-random integration of the HPV genome in cervical cancer. PLoS One 7, e39632 (2012). 22761851 10.1371/journal.pone.0039632 PMC3384597

[b25] Ziegert C. . A comprehensive analysis of HPV integration loci in anogenital lesions combining transcript and genome-based amplification techniques. Oncogene 22, 3977–3984 (2003). 12813471 10.1038/sj.onc.1206629

[b26] Akagi K. . Genome-wide analysis of HPV integration in human cancers reveals recurrent, focal genomic instability. Genome Res 24, 185–199 (2014). 24201445 10.1101/gr.164806.113 PMC3912410

[b27] Yaginuma Y. & Westphal H. Analysis of the p53 gene in human uterine carcinoma cell lines. Cancer Res 51, 6506–6509 (1991). 1660340

[b28] Iwasaka T. . Correlation between HPV positivity and state of the p53 gene in cervical carcinoma cell lines. Gynecol Oncol 48, 104–109 (1993). 8380785 10.1006/gyno.1993.1016

[b29] Olivier M., Hollstein M. & Hainaut P. TP53 mutations in human cancers: origins, consequences, and clinical use. Cold Spring Harb Perspect Biol 2, a001008 (2010). 20182602 10.1101/cshperspect.a001008 PMC2827900

[b30] Ishida G. M. . Small cell neuroendocrine carcinomas of the uterine cervix: a histological, immunohistochemical, and molecular genetic study. Int J Gynecol Pathol 23, 366–372 (2004). 15381906 10.1097/01.pgp.0000139637.01977.61

[b31] Schrock E. . Multicolor spectral karyotyping of human chromosomes. Science 273, 494–497 (1996). 8662537 10.1126/science.273.5274.494

