# Supplementary material for: HPV positive neuroendocrine cervical cancer cells are dependent on Myc but not E6/E7 viral oncogenes
Source: Sci Rep. 2017 Apr 5;7:45617. doi: 10.1038/srep45617 (PMC5381214; doi:10.1038/srep45617)
Supplement: Supplementary Information [file srep45617-s1.pdf]

Derivation of HPV positive neuroendocrine cervical cancer cells which are dependent on Myc but not E6/E7 viral oncogenes

Hang Yuan*[1], Ewa Krawczyk*[1], Jan Blancato[1], Christopher Albanese[1,2], Dan Zhou[1], Naidong Wang[1], Siddartha Paul[1], Faris Alkhilaiwi[1,3], Nancy Palechor-Ceron[1], Aleksandra Dakic[1], Shuang Fang[1], Sujata Choudhary[1], Tung-Wei Hou[1], Yun-Ling Zheng[2], Bassem R. Haddad[2], Yukari Usuda[1], Dan Hartmann[1], David Symer[4], Maura Gillison[5], Seema Agarwal [1], Danny Wangsa[6], Thomas Ried[6], Xuefeng Liu[1], Richard Schlegel[1]

Department of Pathology[1] and Oncology[2], Georgetown University Medical School, Washington DC, 20057; [3]College of Pharmacy, King Abulaziz University, Jeddah, Saudi Arabia; [4]Human Cancer Genetics Program and Dept. of Molecular Virology, Immunology and Medical Genetics, Ohio State University Comprehensive Cancer Center, [5]Dept. of Internal Medicine, Ohio State University Comprehensive Cancer Center, Columbus, OH 43210; [6]Cancer Genomics Section, Center for Cancer Research, National Cancer Institute, Bethesda, MD 20892

Supplemental Material

Methods

STR analysis: Short tandem repeat (STR) analysis (ie, DNA fingerprinting) was performed using a commercially available kit (Cell ID System; Promega Corporation, Madison, WI). This system allows the co-amplification and three-color detection of 10 loci (nine *STR* loci and the Y-chromosome–specific Amelogenin). This approach provides a powerful level of discrimination of approximately 2.92×10^9. The following *STR* markers were tested in addition to the Amilogenin locus: *CSF1PO*, *TPOX*, *TH01*, *vWA*, *D21S11*, *D16S539*, *D7S820*, *D13S317*, and *D5S818*. The PCR amplification was performed according to the manufacturer's recommended protocol. Detection of the amplified fragments was achieved with the ABI 3100 genetic analyzer (Applied Biosystems). Data analysis and allele size determination were performed using GeneMapper Software (Applied Biosystems).

Cell cycle assay: Cells were fixed with Vindalov citrate buffer, stained with propidium iodide, and analyzed by use of a FACSStar Plus dual-laser system (Becton Dickinson).

Invasion assay: The invasion of GUMC-395 cell line was monitored and measured using an established protocol for the xCELLigence Real-Time Cell Analyzer Dual Purpose (RT-CA DP) instrument (ACEA Biosciences). Briefly, E- plates 16 (ACEA Biosciences) were coated with 1mg/ml collagen type I and then rinsed with phosphate buffered saline (DPBS, Invitrogen). Human umbilical vein endothelial cells (HUVEC, 3.5×10^4/well; Lonza) were plated and incubated for 24-31 hours in endothelial growth medium (EGM-2; Lonza). Next, EGM-2 medium was substituted with FY medium containing 1×10^4 GUMC-

395 cells per well. The plate was incubated and the experiment was run for 10-12 hours. The results were analyzed with RT-CA software (ACEA Biosciences).

Migration assay: GUMC-395 cells were starved overnight with serum-free F medium containing 10µM Y-27632. Next day, the bottom chambers of a RT-CA CIM-16 plate (ACEA Biosciences) were filled with 160µl serum containing FY medium, top chambers with 50µl serum free-FY and the background was read. Then, 100 µl of serum-free FY medium containing $1x10^5$/chamber GUMC-395 cells was added to the top chambers and the experiment was run for 24 hours. Results were analyzed using the RT-CA software (ACEA Biosciences).

Figure Legend

Suppl. Figure 1: The timeline indicates the patient's clinical history, therapy and disease status.

Suppl. Figure 2. Migration assay. The migration of GUMC-395 cells in growth medium was monitored and measured for up to 50 hours. The tested cell line shows a very high level of migratory activity, in comparison to non-tumorigenic HEC cells.

Suppl. Figure 3. Invasion assay. The invasion of GUMC-395 cell line was monitored and measured using incubation with human umbilical vein endothelial cells (HUVEC). The experiment was run for up to 5 days. GUMC-395 cells line shows a high level of invasion

activity, in comparison to normal human ectocervical cells (HEC), as well as medium alone.

Suppl. Figure 4: Array-based CGH ideograms of chromosomal gains and losses. DNA gain is indicated by bars on the right of the tracings and DNA loss by bars on the left. The length of the bar delineates the region of the chromosome involved in the gain or loss. The results show several folds amplification of the *MYC* gene on 8q24.21 (green arrow) and the loss of *P53* gene on 17p13.1 (blue arrow).

Suppl. Figure 5. Cell cycle assay. Cells for cell cycle analysis were fixed with Vindalov citrate buffer, stained with propidium iodide, and analyzed by use of a FACSStar Plus dual-laser system. The GUMC-395 cell line appears diploid.

Suppl. Figure 6. SiHa cell growth inhibited by E7 siRNA in medium with ROCK Inhibitor. The growth of SiHa cells was monitored for 96 hours, and cell numbers were counted at 0, 1, 2, 3 and 4 days post transfection.

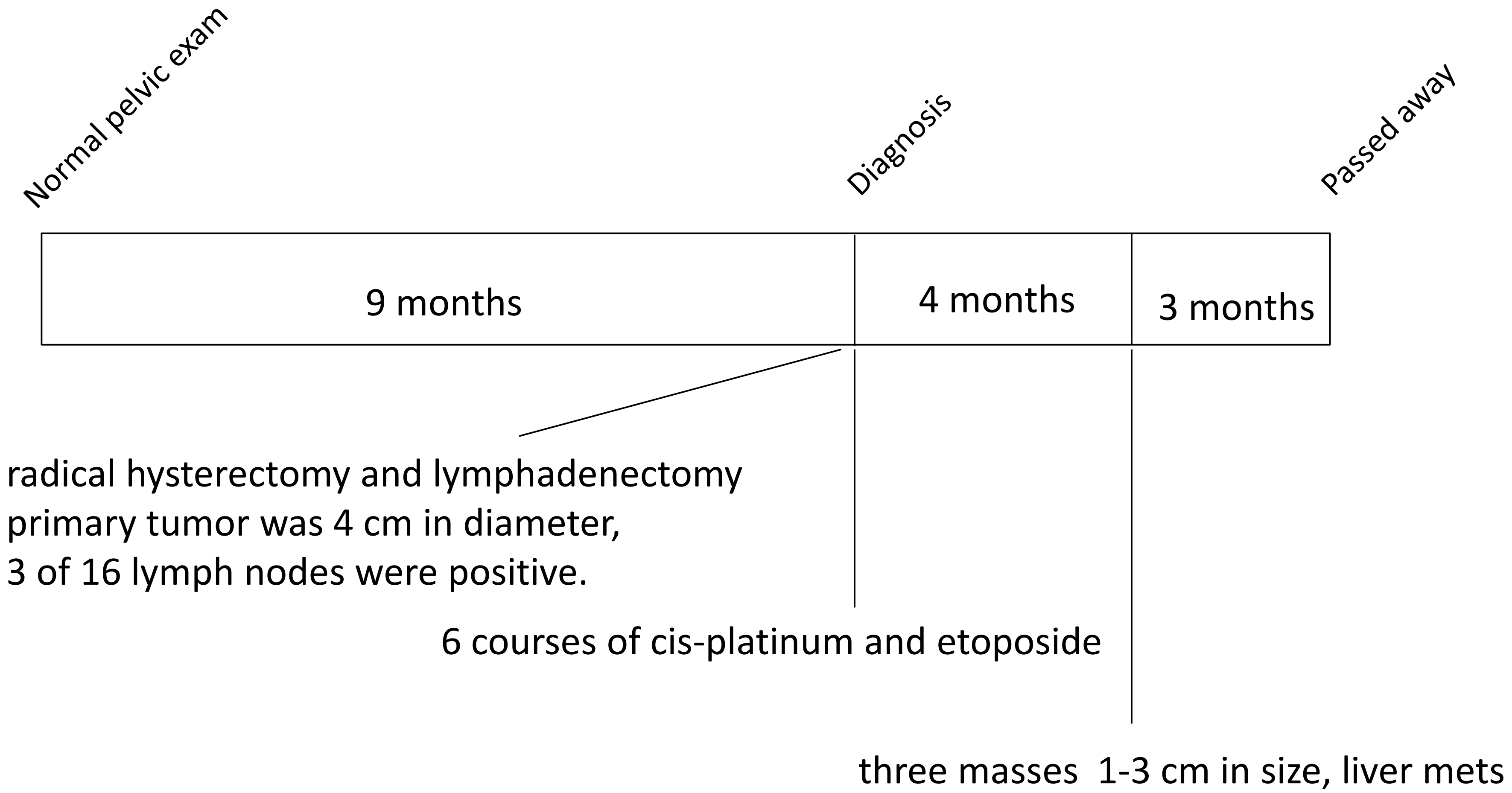

Suppl. Figure 1: The timeline indicates the patient’s clinical history, therapy and disease status.

coating	feeder	medium	Y-27632	growth
no	yes	F medium	yes	+
no	no	F medium	yes	++
no	no	conditioned medium	no	+
no	no	conditioned medium	yes	+
no	no	Keratinocytes medium	yes	+
no	no	DMEM medium	no	+
no	no	Stem cell medium mTeSR™1	no	+
gelatin	no	F medium	yes	++
matrigel	no	Stem cell medium mTeSR™1	no	+++
collagen	no	F	no	+ (low attachment)
collagen	no	F	yes	++++

Supplemental Table 1. Cell growth condition

Loci	Lymphocyte	395/P7	395/P28
AMEL	X	X	X
CSF1PO	10,11	10,11	10,11
D13S3317	11,14	11,14	11,14
D165539	11,12	11,12	11,12
D21S11	24.2,31.2	24.2,31.2	24.2,31.2
D5S818	12	12	12
D7S820	8,10	8,10	8,10
TH01	9.3	9.3	9.3
TPOX	8,10	8,10	8,10
vWA	15,17	15,17	15,17

Supplemental Table 2. STR (fingerprinting) analysis of the patient's lymphocytes and the early and late passage tumor cell cultures

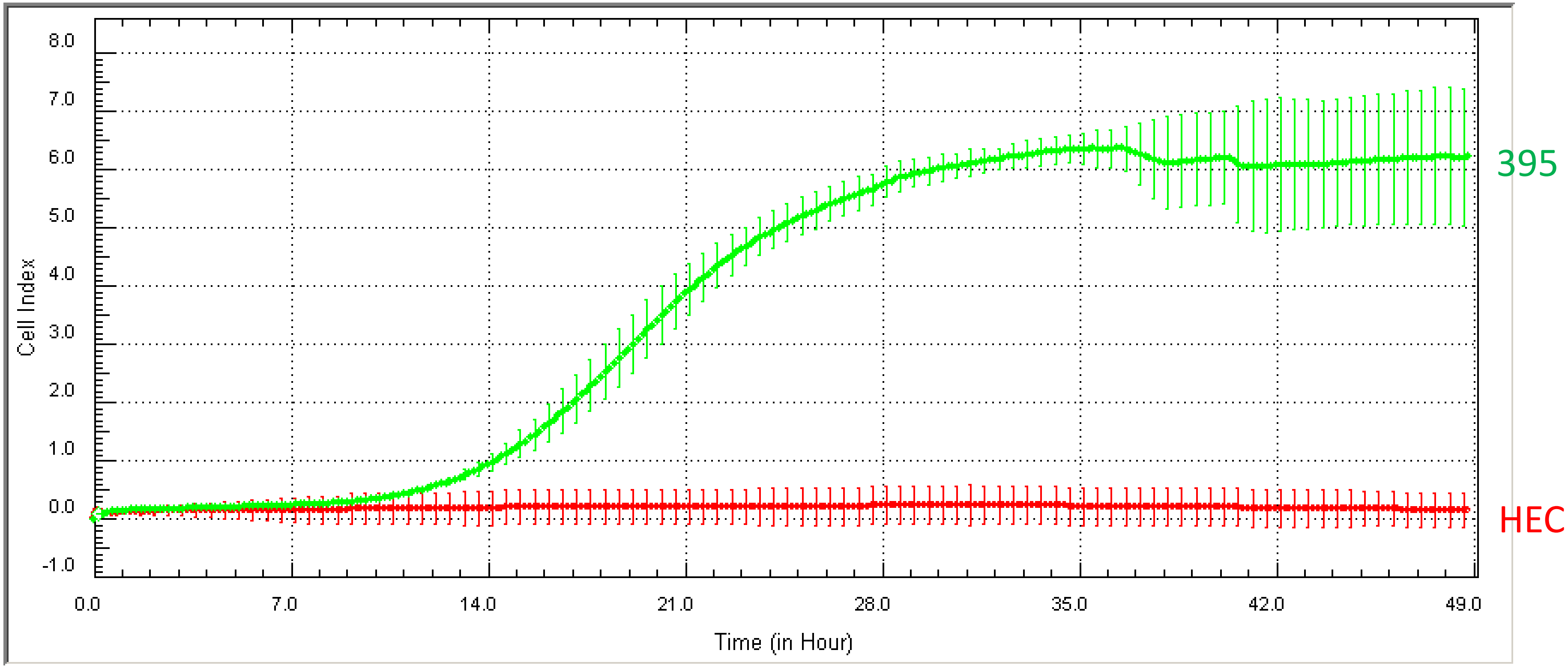

Supplemental Figure 2. Migration assay. The migration of GUMC-395 cells in growth medium was monitored and measured for up to 50 hours. The tumor cell line 395 shows a very high level of migratory activity, in comparison to non-tumorigenic control HEC cells.

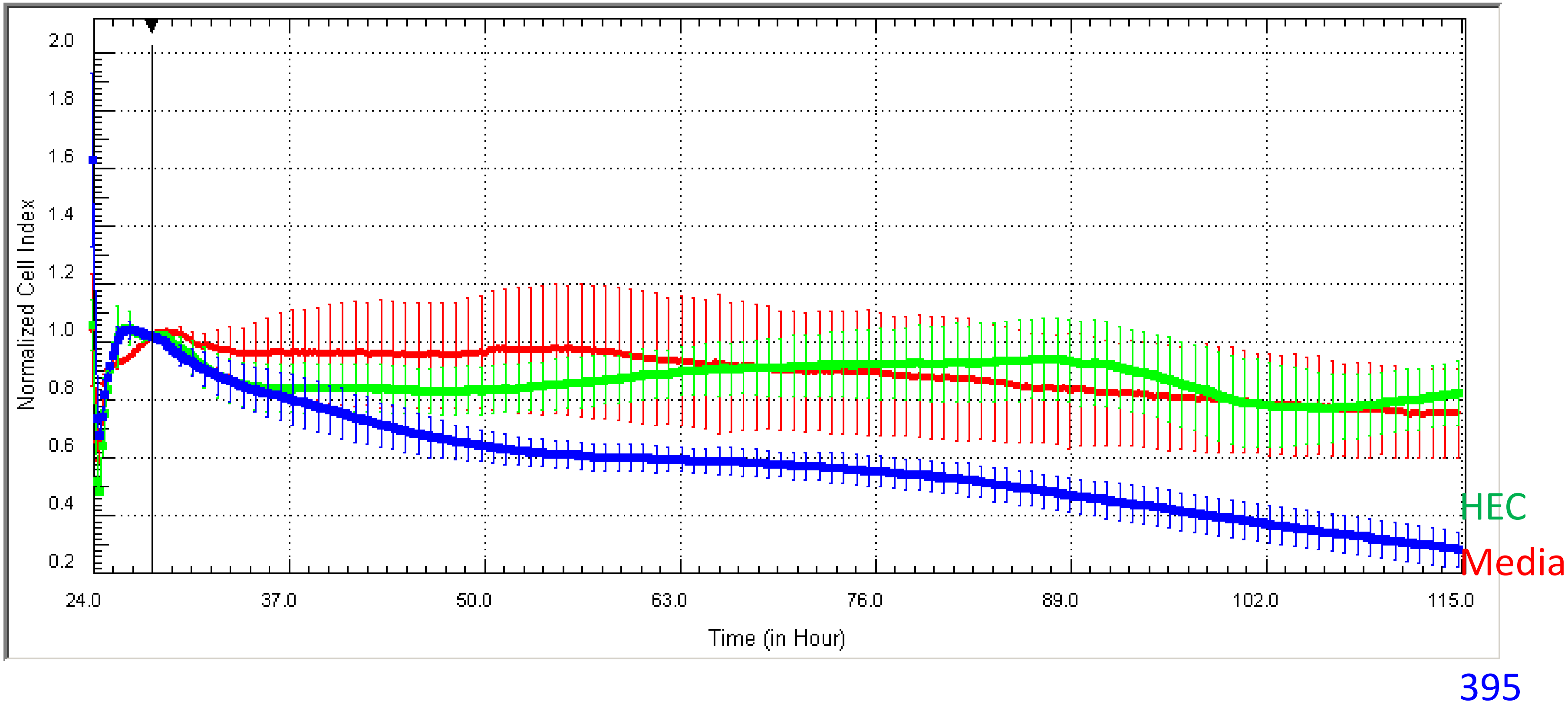

Supplemental Figure 3. Invasion assay. The invasion of GUMC-395 cell line was monitored and measured using incubation with human umbilical vein endothelial cells (HUVEC). The experiment was run for up to 5 days. GUMC-395 cells line shows a high level of invasion activity, in comparison to normal control human ectocervical cells (HEC) and medium control.

	tumor	Cell line GUMC-395
Ki67	93% (IHC)	Very high (IHC)
HPV type	HPV16 (RNA-seq)	HPV16(PCR typing, Realtime PCR)
p53	R273C mutation (RNA-seq)	R273C mutation (Sanger sequencing)
Myc	Myc amplification (RNA-seq)	Myc amplification (Real time PCR)
Chromogranin A	High expression (IHC)	High expression (IHC)
Synaptophysin	High expression (IHC)	High expression (IHC)
Somatostatin receptor-2	High expression (IHC)	High expression (IHC)

Supplemental Table 3. Comparisons between tumor and derived neuroendocrine cell GUMC395.

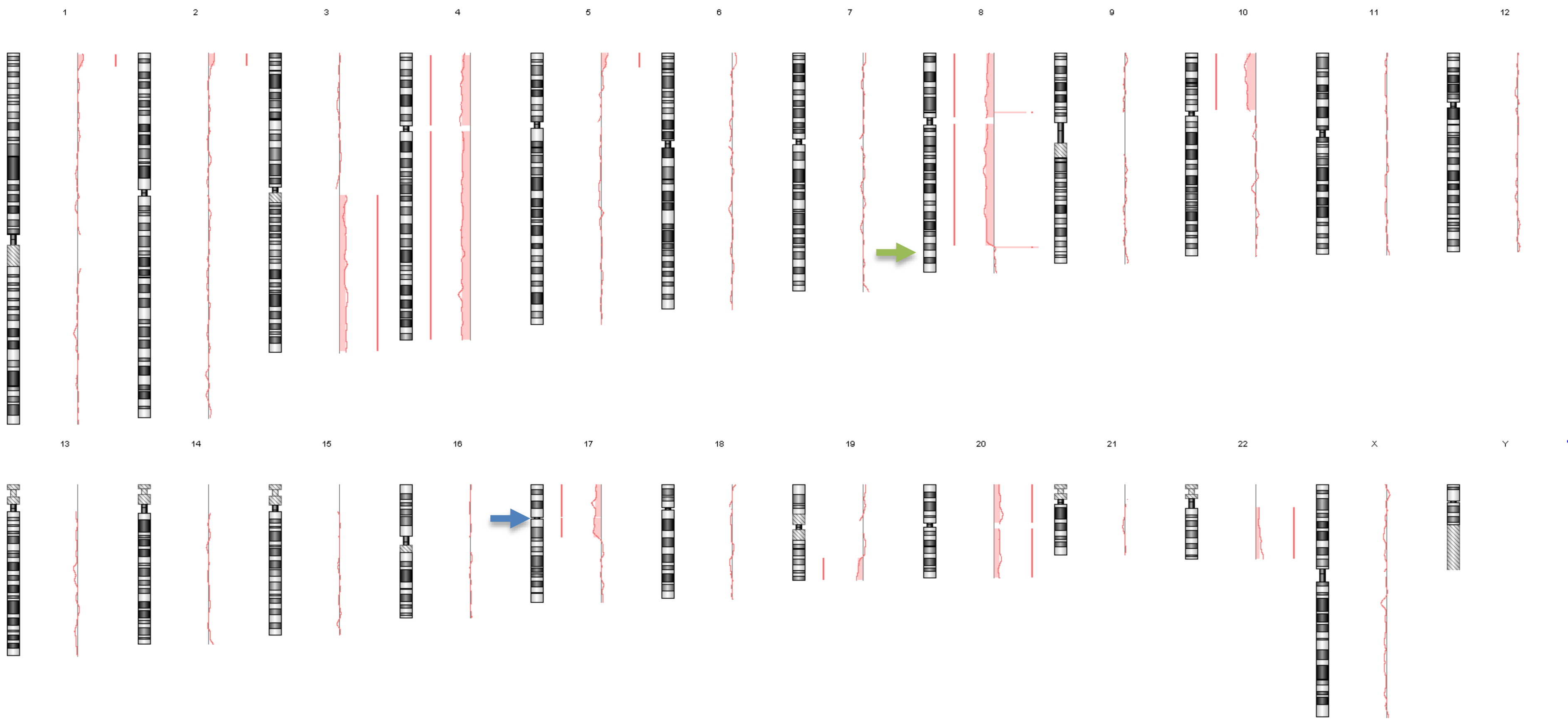

Supplemental Figure 4. Array-based CGH ideograms of chromosomal gains and losses. DNA gain is indicated by bars on the right of the tracings and DNA loss by bars on the left. The length of the bar delineates the region of the chromosome involved in the gain or loss. The results show several folds amplification of the *MYC* gene on 8q24.21 (green arrow) and the loss of *P53* gene on 17p13.1 (blue arrow).

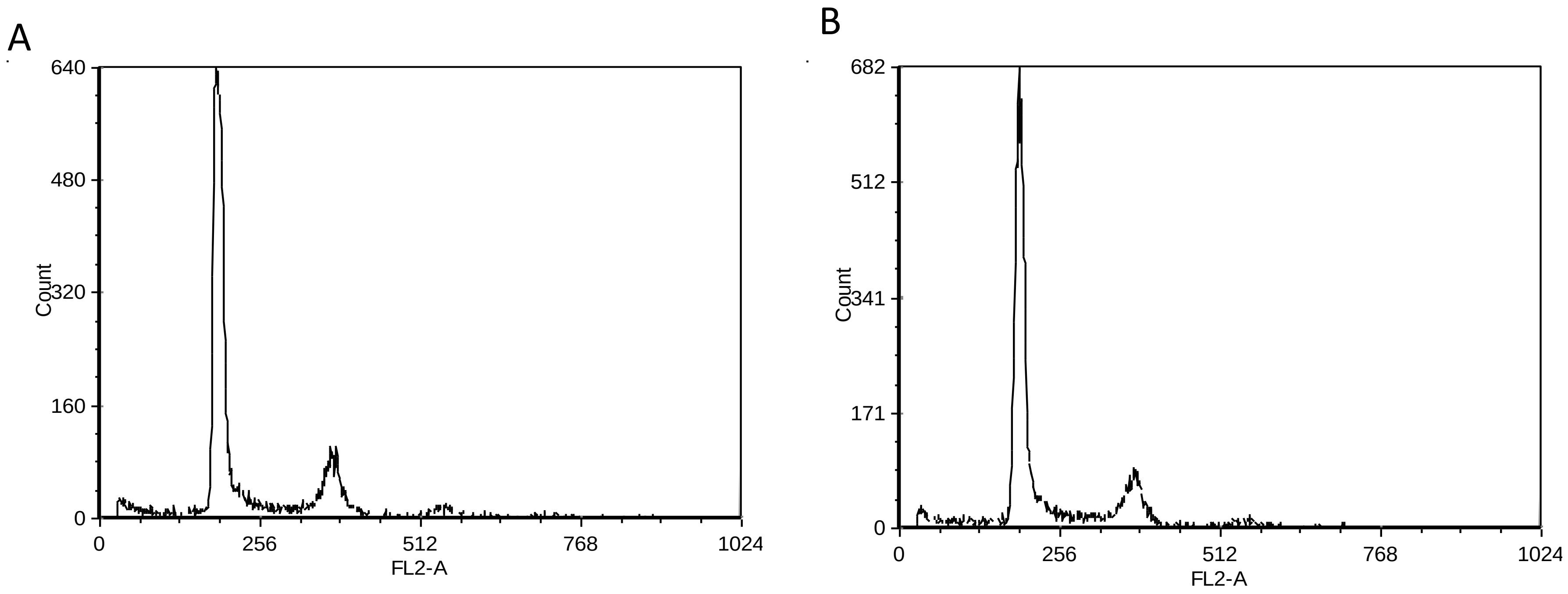

Supplemental Figure 5. Cell cycle assay. Cells for cell cycle analysis were fixed with Vindalov citrate buffer, stained with propidium iodide, and analyzed by use of a FACSStar Plus dual-laser system. (A) Normal primary human foreskin keratinocyte as a diploid control. (B) The GUMC-395 cell line appears diploid.

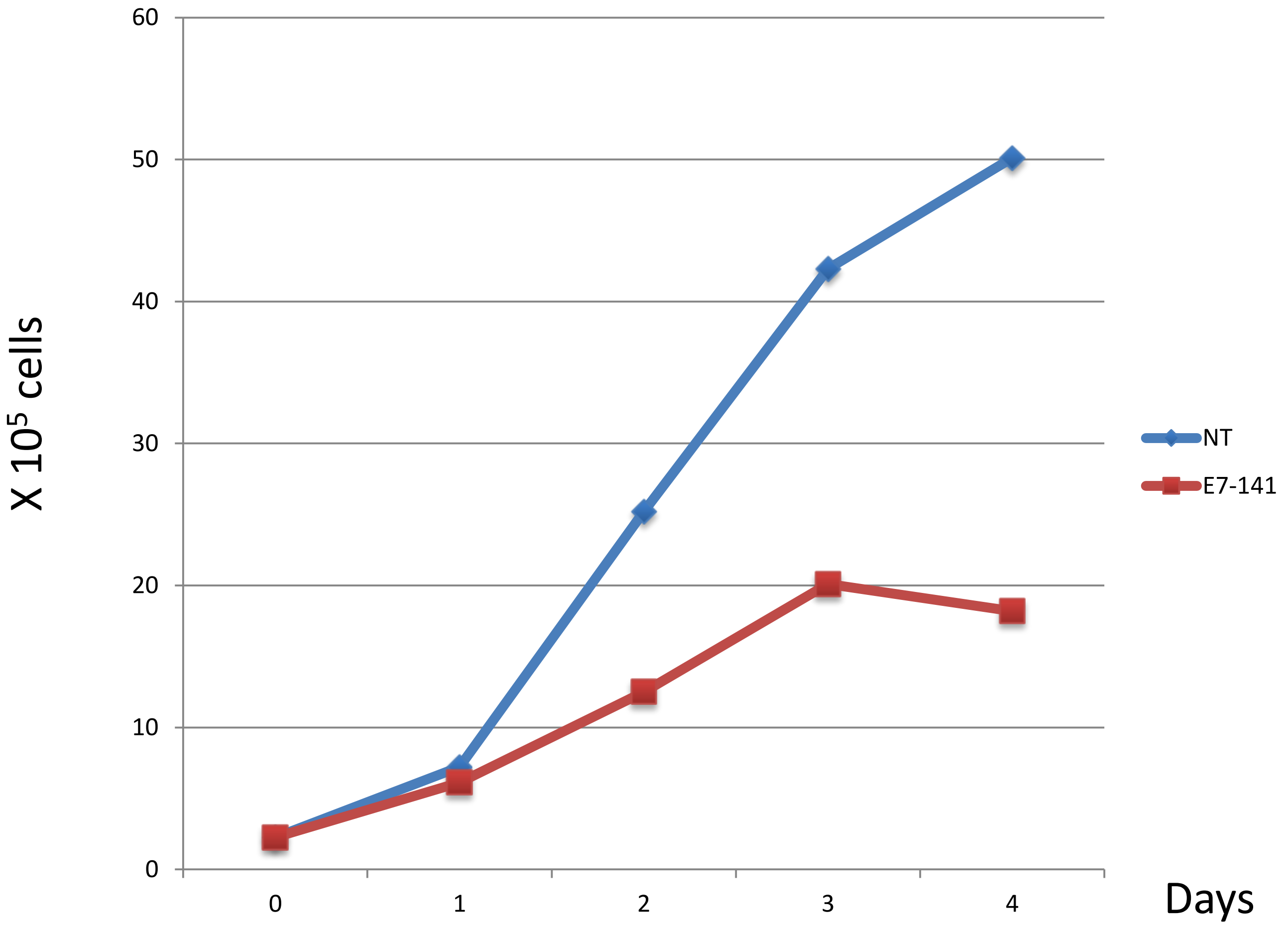

Supplemental Figure 6. SiHa cell growth inhibited by E7 siRNA in medium with ROCK Inhibitor. SiHa cells were tranfected with control non-target siRNA or E7 siRNA. The growth of SiHa cells was monitored for 96 hours, and cell numbers were counted at 0, 1, 2, 3 and 4 days post transfection.
